# Considerations for a Retained Foreign Body in the Posterior Orbital Apex

**DOI:** 10.7759/cureus.19228

**Published:** 2021-11-03

**Authors:** Gabriella Schmuter, Ethan M Stern, Michelle Packles

**Affiliations:** 1 Ophthalmology, BronxCare Health System, Bronx, USA

**Keywords:** open globe, trauma, foreign body, plastic surgery, oculoplastics, ophthalmology

## Abstract

Orbital foreign bodies must be approached with complex considerations involving their precise location and composition in order to adequately guide management. Physicians must carefully weigh the advantages and risks of surgical and medical intervention compared to conservative management. We present a case of a male patient with penetrating trauma to the eye that resulted in open globe injury and orbital foreign body, presumed metallic, at the posterior orbital apex near the optic nerve. As such, despite the uncertainty of the exact composition of the object, the medical team and patient agreed to conservative management given its high-risk anatomical location.

## Introduction

An orbital foreign body is defined as any foreign object either partly or completely within the orbit, regardless of whether or not the object penetrates the globe. Orbital foreign bodies are further classified as purely orbital or transorbital, in which case it involves an adjacent region such as the intracranial or paranasal sinus space. Typically, orbital foreign bodies are secondary to trauma. Rarely, they may be iatrogenic, such as secondary to surgical orbital implants. As such, patients typically present with a history of traumatic injury, and the majority of patients have been found to be young men (approximately ages 15 to 37) [1-3].

Orbital foreign bodies have the potential to lodge within the walls of the orbit, and may subsequently damage nearby structures, such as the globe, cranial nerves (particularly, cranial nerves II, III, IV, V, and VI), and extraocular muscles. Though considered rare, an orbital foreign body may penetrate directly through the globe and lodge in the orbital apex, as we present in this case. The symptoms of a patient with an orbital foreign body vary widely and may occasionally be asymptomatic as an occult foreign body. Patients may present with visual disturbances, pain, swelling, or double vision.

Foreign bodies may be composed of inorganic material, such as steel, plastic, glass, or copper, or organic, such as wood or vegetative material. If a metallic intraocular foreign body is small in size, in the deep orbit, and composed of inert material, it may be left in place. However, some metallic objects, namely copper, steel, lead, and iron, are not inert. In such cases, complete surgical removal should be attempted due to the risk of infection and inflammation. If an open globe injury is present, the globe should be repaired promptly. When an open globe trauma has an orbital foreign body that involves the posterior segment as opposed to the anterior segment, rates of complications and need for re-operation are higher [3,4]. An open globe should be ideally repaired within 24 hours of trauma, and removal of an orbital foreign body should ideally occur at the same time if possible. Surgical intervention subsequent to globe exploration and repair is recommended if the patient has a neurologic compromise, restriction of eye movements, or development of infection from the orbital foreign body [5].

Nevertheless, if a foreign body is in the posterior orbit, the surgical intervention itself may pose the risk of structural damage. If a patient is managed conservatively, regular monitoring for the development of an abscess or fistula [6] from the intraocular foreign body should be performed. Complications of the retained intraocular foreign body include abscess formation [7], sinus infection, inflammation, fibrosis, migration or spontaneous extrusion, optic neuropathy, or gaze-evoked amaurosis [8]. As the medical team chose to pursue observation and monitoring of our patient, longitudinal tolerance of metallic orbital foreign bodies adjacent to the optic nerve has been appreciated in previous cases [9].

In many cases, the physician must weigh the dilemma of the risk of surgery and the risks of infection and inflammation from a retained foreign body. The outcomes of previous case reports [10-12] demonstrate the delicate balance required in the decision-making process for the management of these traumas. We present a complicated case of an orbital foreign object (presumed metallic) with open globe injury secondary to penetrating trauma that was managed with observation given the object’s location in the posterior apex. This case underscores the challenge of discerning situations that may warrant observation despite a high risk of inflammation or infection given the object’s composition.

## Case presentation

**Figure 1:**
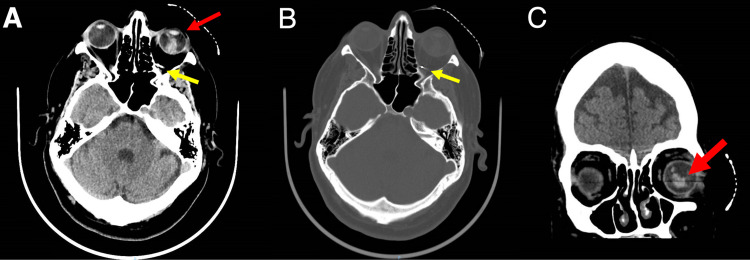
CT of orbits and brain without contrast in the axial (A and B) and coronal (C) planes from the emergency department demonstrating a penetrating open globe trauma (red arrows) along with an orbital foreign body near the orbital apex (yellow arrows).

A 51-year-old patient with a past medical history of daily alcohol use and no past ocular history presented to the St. Barnabas Health System Emergency Department with left eye pain. He states that he was walking in a store when an object “flew into [his] eye,” which caused severe eye pain. The patient believed he had been hit by a bullet ball (BB) gun. The patient's last alcoholic drink was 15 minutes prior to arrival. He denied head injury, fall, numbness, tingling, or contact lens wear. There was a high suspicion of globe rupture of the left eye secondary to a penetrating injury. Computed tomography (CT) imaging of the orbits confirmed the globe rupture and revealed an orbital foreign body near the orbital apex (Figures 1A-1C).

On examination, his visual acuity at near without correction was 20/50 in the right eye, and was found to be hand motion in the left eye. His pupils were round and symmetric without appreciation of an afferent pupillary defect in either eye. His intraocular pressure (IOP) as measured by an iCare tonometer was 9 mmHg in the right eye and 2 mmHg in the left eye. The patient’s extraocular motility was full in both eyes. The examination was unremarkable in the right eye. The left eye demonstrated conjunctival injection. The cornea demonstrated a full thickness wound around the 8 o'clock position with a superficial foreign body in that region. This superficial foreign body was suggestive of glass by gross visual appearance alone. There was no involvement of the limbus. At this time, the patient had an ocular trauma score of 33 [13]. Subsequently, the patient was found to have a positive Seidel sign. Descemet’s folds were present in the left eye. The lens was found to be in place. Corneal edema was present throughout with prolapse of the uvea through the traumatic wound. The anterior chamber demonstrated a 50% hyphema. Details of the iris were not appreciated at the time of this examination. The patient’s dilated fundus exam was deferred in the left until the postoperative period as the priority was to repair the open globe promptly.

Given the high risk of progression toward loss of the eye or infection without immediate treatment, the patient was emergently brought to the operating room for open globe repair and exploration of the left eye. The patient was started on intravenous vancomycin (15 mg/kg) and ceftazidime (50 mg/kg) [14]. In addition, the patient was provided tetanus prophylaxis and ensured adequate pain control.

In the operating room, a speculum was carefully placed to expose the globe, which was found to be hypotonus. An inferonasal, round, and full-thickness corneal defect was noted on the inferonasal cornea. An attempt was made to close the wound with a 10-0 Nylon suture (Bridgewater, NJ: Ethicon) with two crossing slipknots, and the globe subsequently appeared to be watertight. One paracentesis was created at the temporal position and one tract was created at the 11 o'clock position. When the eye was inflated with balanced salt solution, the wound expanded, and it was discovered that there was approximately a 2.0 x 2.0 mm circular full-thickness corneal defect with a full-thickness iris defect posterior to the corneal defect. Ophthalmic viscosurgical devices were used to plug the iris opening to attempt to prevent further prolapse of any vitreous. At this time, a horizontal mattress suture was used to oppose the edges of the wound with three 9-0 Nylon and 8-0 Nylon interrupted sutures (Bridgewater, NJ: Ethicon) in the cornea to oppose the edges. Monofilament sutures are typically used on the cornea due to low tissue reactivity [15]. All viscoelastic was subsequently removed from the eye, and attempts to aspirate the well-formed fibrinous hyphema were made. The two paracentesis wounds were hydrated. Fluorescein was painted on the area of the sutured corneal defect, which was noted to be Seidel negative at this time. The purpose of the Seidel test is to check for leakage of aqueous humor from the anterior chamber [16]. No vitreous was seen exiting the cornea at this time. We injected 0.1 cc of intracameral cefuroxime into the eye. A bandage contact lens, ointment of neomycin, polymyxin-b, and dexamethasone, and an eye patch were placed. The patient was subsequently admitted with a plan for intravenous antibiotics of vancomycin and ceftazidime for two days. 

On his outpatient visit the next day, the patient was started on topical moxifloxacin and prednisolone acetate. His visual acuity continued to be hand motion with full extraocular motility. No appreciable leak was evident at the bedside. Dilated fundus examination was unremarkable at this time with clear optic nerve margins. His IOP postoperatively was 7 mmHg in the left eye as measured by iCare tonometry.

At this time, the orbital foreign body near the orbital apex remained in place. The object was presumed to be of metallic composition given the patient's suggestion that the trauma was due to a BB gun along with the object's appearance on the original CT image from the emergency department. BB gun pellets are typically composed of steel covered in zinc or copper. Given the location and the high risks of surgery, along with an extensive discussion with the patient and his family, the medical team opted for conservative management of the orbital object with careful observation and monitoring. The patient was referred for follow-up with the retina specialist as well to monitor for any postoperative changes in the posterior segment.

## Discussion

When assessing patients who may present with an orbital foreign body, a thorough history and physical must be performed to determine the wound of entry. A history of projectile injury, such as due to an explosion or gunshot, should be evaluated for an orbital foreign body. Physicians should urgently rule out the presence of an open globe injury or traumatic optic neuropathy. In addition, characteristics such as the type of foreign body and depth of penetration are important to note if possible. The surrounding environment at the time of trauma may provide a clue regarding the composition of the foreign body. Thorough evaluation for orbital trauma is essential to avoid a missed diagnosis [11].

The location of the foreign body and the presence of any optic nerve involvement may be ruled out with imaging. A CT scan without contrast of the orbits in axial, coronal, and parasagittal views and brain is the gold standard for imaging of an orbital foreign body after trauma [17,18]. If it is clear that the foreign body is not a metallic ferromagnetic object, additional imaging may be obtained with magnetic resonance imaging (MRI). MRI should be reserved for foreign objects that are highly suspicious to have the composition of wood [19-21] or organic material or for fragments that are less than 0.5 mm on CT scan. Of note, patients who are managed conservatively with the retained metallic intraocular foreign body should not undergo MRI as the object may be ferromagnetic. Ferromagnetic objects under MRI may become unrestrained and airborne into the scanner's magnet due to the forces exerted during the imaging process [22]. A contact ultrasound (B-scan) of the eye may be used prior to CT for suspected intraocular involvement, though results are highly operator-dependent and can potentially risk extrusion of intraocular contents. B-scan ultrasound is helpful to detect the presence of a retinal detachment or hemorrhage in cases of trauma. If the patient presents with a reliable history and palpable foreign body, plain x-ray radiographs may be adequate for diagnosis. However, plain radiographs typically provide less detail of intraocular foreign bodies compared to CT scans, particularly if the foreign body is composed of graphite, plastic, or wood. Orbital foreign bodies composed of aluminum are particularly challenging as they are not well-appreciated either x-ray radiographs or CT scans [23].

Modern imaging techniques have provided advantages for increasing the accuracy of surgical navigation [24]. In particular, image-guided CT with real-time imaging has been successfully used to endoscopically remove foreign objects near the optic nerve [25]. In addition, interdisciplinary approaches with otolaryngology have been successful in endoscopic removal of intra-orbital metallic foreign bodies through a transnasal approach [26]. Nevertheless, such a surgical approach carries its own inherent risks including inadvertent damage to the optic nerve and nearby structures [27].

In addition to considerations regarding the risks and benefits of surgical intervention for an orbital foreign body, patients are typically provided with tetanus prophylaxis upon presentation based on vaccination status. If there is a history of trauma or signs of infection, patients should be placed on broad-spectrum antibiotics, with a potential coverage of anaerobic microbes or fungus. Antibiotics with strong blood-brain-barrier penetration are ideal given the proximity of the orbit to the central nervous system. Typically, a third-generation cephalosporin and vancomycin are provided for patients with suspected globe perforation, rupture, or complication of the trajectory of the intraorbital foreign body, such as intracranial infection. 

Anteriorly located orbital foreign bodies without penetration of the globe are associated with a better prognosis, especially with regard to visual acuity [3,4]. Foreign bodies composed of organic materials pose an increased risk of causing endophthalmitis or infections of the central nervous system. This is due to the fact that the bacteria from organic foreign bodies cause rapid onset inflammation that can quickly impact the optic nerve [28,29]. Comparatively, all intraocular surgical procedures pose an inherent risk of complications, including retinal detachment, endophthalmitis, and vitreoretinopathy. Orbital foreign bodies due to BB gun pellets with lead composition have been rarely associated with the development of traumatic chorioretinitis sclopetaria [30]. Given the risk of retinitis and the risk of postoperative complications, the patient was advised to follow up closely with a retina specialist. 

It is important for patients to be aware of preventative interventions regarding trauma with an orbital foreign body, particularly patients who are young adults or children [31]. Parents of young children must ensure caution with children and projectiles, such as toy guns, dart guns, and BB guns. In industrial and sports settings, appropriate eye protection is paramount to minimize risk. 

## Conclusions

The management of orbital foreign bodies is complex given a variety of considerations that guide management. Each option weighs surgical, medical, or conservative management and decisions made between physicians and patients culminate in a variety of risks and benefits. In this case, the medical team opted for conservative management and referred to the retina team for intraocular complications secondary to the patient’s globe trauma, including the presence of proliferative vitreoretinopathy. Children and individuals who participate in activities, industries, occupations, or sports that involve projectile objects must always wear adequate eye protection to avoid orbital foreign body injury.
